# Systematic development of a set of implementation strategies for transitional care innovations in long-term care

**DOI:** 10.1186/s43058-023-00487-3

**Published:** 2023-08-28

**Authors:** Amal Fakha, Bram de Boer, Jan. P. Hamers, Hilde Verbeek, Theo van Achterberg

**Affiliations:** 1 Department of Health Services Research, Maastricht University, CAPHRI Care and Public Health Research Institute, Maastricht, the Netherlands; 2 Living Lab in Ageing and Long-Term Care, Maastricht, the Netherlands; 3 KU Leuven, Department of Public Health and Primary Care, Academic Centre for Nursing and Midwifery, Leuven, Belgium

**Keywords:** Implementation Mapping, Implementation strategy, Innovation, Intervention, Transitional care, Factors, Older persons, Long-term care

## Abstract

**Background:**

Numerous transitional care innovations (TCIs) are being developed and implemented to optimize care continuity for older persons when transferring between multiple care settings, help meet their care needs, and ultimately improve their quality of life. Although the implementation of TCIs is influenced by contextual factors, the use of effective implementation strategies is largely lacking. Thus, to improve the implementation of TCIs targeting older persons receiving long-term care services, we systematically developed a set of viable strategies selected to address the influencing factors.

**Methods:**

As part of the TRANS-SENIOR research network, a stepwise approach following Implementation Mapping (steps 1 to 3) was applied to select implementation strategies. Building on the findings of previous studies, existing TCIs and factors influencing their implementation were identified. A combination of four taxonomies and overviews of change methods as well as relevant evidence on their effectiveness were used to select the implementation strategies targeting each of the relevant factors. Subsequently, individual consultations with scientific experts were performed for further validation of the process of mapping strategies to implementation factors and for capturing alternative ideas on relevant implementation strategies.

**Results:**

Twenty TCIs were identified and 12 influencing factors (mapped to the Consolidated Framework for Implementation Research) were designated as priority factors to be addressed with implementation strategies. A total of 40 strategies were selected. The majority of these target factors at the organizational level, e.g., by using structural redesign, public commitment, changing staffing models, conducting local consensus discussions, and organizational diagnosis and feedback. Strategies at the level of individuals included active learning, belief selection, and guided practice. Each strategy was operationalized into practical applications.

**Conclusions:**

This project developed a set of theory and evidence-based implementation strategies to address the influencing factors, along further tailoring for each context, and enhance the implementation of TCIs in daily practice settings. Such work is critical to advance the use of implementation science methods to implement innovations in long-term care successfully.

**Supplementary Information:**

The online version contains supplementary material available at 10.1186/s43058-023-00487-3.

Contributions to the literature

- ▪ This original work provides a set of theory and evidence-based implementation strategies selected specifically to improve the implementation of transitional care innovations.
- ▪ This set of strategies serves as an invaluable and practical guide for future implementers of transitional care innovations in long-term care practice.
- ▪ The application of Implementation Mapping method to select strategies for implementing transitional care innovations was not conducted beforehand.
- ▪ The used methodology, Implementation Mapping, can be replicated to improve the implementation of other innovations in long-term care services.

## Background

Since the early 2000s, the concept of transitional care, defined by Naylor et al. (2011, page 747), as “a broad range of time-limited services designed to ensure health care continuity, avoid preventable poor outcomes among at-risk populations, and promote the safe and timely transfer of patients from one level of care to another or from one type of setting to another,” has gained momentum [1, 2]. Moreover, improving care transitions for highly vulnerable and chronically ill older persons during their multiple movements between different care settings (i.e., older persons 65 years and above receiving long-term care services in multiple care settings — focus of this paper) is emerging as the face of delivering exemplary modern-day long-term care.

In alignment with this, the development of a wide range of transitional care innovations (TCIs) flourished as a potential solution to minimize the care fragmentation and adverse events associated with poor care transitions [3–5]. To date, the literature indicates that at least 55 different TCIs were implemented covering multiple care pathways (e.g., hospital to home, home to nursing home, hospital to transfer unit to home) and targeting older persons with various chronic conditions (e.g., stroke, dementia, heart failure) [3, 5–10]. Some of these TCIs showed possible beneficial effects such as reducing hospital readmissions, preventing emergency department visits, avoiding unnecessary admission to a nursing facility, enhancing treatment adherence, or improving the quality of life for older persons [9, 11–13].

While the aims of many TCIs are diverse, there are similarities among their components as well as ambiguity on how they are implemented in real practice. Accordingly, a rising universal awareness exists among healthcare professionals, scientists, and policy-makers, that despite the evidence of the benefit of healthcare innovations such as TCIs, their implementation is hardly straightforward [14]. Specifically, the implementation of TCIs is often complex and influenced by an array of factors (barriers, facilitators) [3]. A lack of organizational resources, low feasibility of implementing the TCI within an organization, and variable staff commitment are among the common barriers [3]. Whereas the presence of staff with transitional care roles (e.g., transition care nurse, transition care manager), supportive organizational leadership, and strong engagement of key stakeholders are all facilitating factors to implement a TCI [3, 15]. Hence, there is a need to use effective implementation strategies, defined as “methods or techniques used to improve the adoption, implementation, sustainment, and scale-up of evidence-based health interventions into usual care” [16, 17] to address the influencing factors. This will help foster the implementation of TCIs into practice to ensure that older persons receive the expected benefits [14, 18]. To promote the use of implementation strategies, several taxonomies and compilations of strategies were developed to help implement interventions in healthcare in a successful way [19–23].

Few studies that implemented TCIs reported on factors (barriers, facilitators) that influenced the implementation [7, 24, 25]. Moreover, they hardly indicated if implementation strategies were used, and if so, what they were. On the other hand, although the selection of implementation strategies to use in implementing TCIs is starting to pick up as indicated in recent studies, it remains vague how implementers of innovations select strategies to improve the implementation [10, 26]. Consequently, TCIs tend to be more likely implemented in a manner of either “this had worked in the past,” “this is known to work,” “this seems promising,” or “this is how we have always done it.” Furthermore, implementation strategies that were used and effective in certain studies are usually selected and copied for use in subsequent studies, despite differences in the intervention itself, the recipients, and the context where they are implemented. Hence, this approach will probably lead to limited success in implementing TCIs [16]. Therefore, two problems arise. First, implementation strategies are context-specific, and what works in one context might not work in another. Second, implementation strategies should be linked to the implementation factors in the relevant context as well as selected based on both mechanisms of change that explain how factors can be addressed and on available evidence for their effectiveness [27].

While the literature provides several taxonomies or overviews of theory-based or expert-recommended implementation strategies, some of which provide linkages to implementation factors as well as some guidance on selection, feasibility, and importance of strategies, these stem mostly from fields of implementing interventions in general healthcare [20, 22, 23, 28–30]. To date, there is an absence of a set of strategies developed for implementing TCIs specifically to enhance care transitions for older persons receiving long-term care services in multiple care settings (e.g., nursing homes, assisted living facilities, homecare). This paper aims to describe a novel and systematic development of a set of strategies to improve the implementation of TCIs and increase their chances of success. This work is based on integrating findings on barriers to and facilitators of TCIs’ implementation from previous studies by the research team and others [3, 15, 31], to then propose linkages to and the application of implementation strategies to address or leverage these factors.

The overall goal of this project is to guide future implementers of TCIs (i.e., scientists, health care professionals, and leaders of long-term care organizations) and help minimize the gap between insights on optimal transitional care from existing TCIs and the limited use of these insights in practice.

## Methods

This project followed a stepwise approach informed by the Implementation Mapping methodology to develop, choose, and design a carefully selected set of implementation strategies specifically for implementing TCIs [32]. We formed a working group to perform the different steps consisting of the core research team (AF, BdB, TvA; holding expertise in both transitional care and implementation science) and one additional expert in the Implementation Mapping technique. The Maastricht University Faculty of Health, Medicine, and Life Sciences Ethics Committee approved this work (approval no. FHML-REC/2022/003). This paper followed the Standards for Quality Improvement Reporting Excellence (SQUIRE) guidelines for reporting new knowledge about how to improve healthcare [33] (see Additional file 1).

### Rationale for using Implementation Mapping

Based on combining aspects from both implementation science and Intervention Mapping, Implementation Mapping provides five consecutive tasks to develop, select, and design implementation strategies [32]. Implementation Mapping incorporates theory and evidence and provides a systematic way to address the key implementation factors by linking them to the relevant change methods to guide successful implementation. For the scope of this project, we applied iteratively only the first three tasks of Implementation Mapping: (1) *conduct a needs assessment*; (2) *identify implementation outcomes, performance objectives, determinants, and change objectives*; (3) *select theoretical change methods and design implementation strategies*, to develop implementation strategies for TCIs (see Table 1).

**Table 1:** Overview of steps, objectives, and methods performed

Steps	Objectives	Methods
**Preliminary step**	▪ Describe TCIs and identify their core components	▪ Literature review by the research team [3] ▪ Analysis and mapping to transitional care core components proposed by Naylor et al. [34]
**1. Conduct a needs assessment**	▪ Determine the priority implementation factors that influence the implementation of different types of TCIs	▪ Compilation of findings from three previous studies by the research team (literature review, Delphi study, collective case study) [3, 15, 31]
**2. Identify implementation outcomes, performance objectives, determinants, and change objectives**	▪ Identify underlying changeable determinants (theoretical constructs) for the implementation factors from step 1 ▪ Determine the TCIs’ implementation outcomes ▪ Specify performance objectives ▪ Develop matrices of change objectives based on performance objectives and determinants of implementing TCIs	▪ Data from the preliminary step and needs assessment step ▪ Input from the expert on Implementation Mapping
**3. Select theoretical change methods and design implementation strategies**	▪ Identify and select theoretical change methods (strategies) ▪ Retrieve empirical evidence on the effectiveness of strategies ▪ Validate and refine the final selection of strategies ▪ Develop practical applications for selected strategies	▪ Literature on theoretical change methods (published behavioral change taxonomies and compilations of implementation strategies) ▪ Literature scan for evidence on the effectiveness of strategies ▪ Input from experts in the fields of implementation science, transitional care interventions, and long-term care (four consultation sessions)

*TCIs* Transitional care innovations

As a *preliminary step* and prior to performing tasks 1–3 in Implementation Mapping, we opted to first describe what TCIs and their core components are, in order to clarify what the innovation to be implemented is for future users of the implementation strategies. Hence, we utilized the findings from a scoping review by the research team that identified different TCIs and their specific key elements (e.g., case management, follow-up visits after a transition, and appointing a transitional care nurse) [3]. For each TCI, we mapped its elements to eight core transitional care components: patient engagement, caregiver engagement, patient education, caregiver education, complexity management, care continuity, wellbeing, and accountability; as defined by Naylor et al. (2017) to achieve a holistic care process [34]. Consequently, the elements of the TCIs belonging under each core component were combined. Three of the research team (AF, BdB, TvA) performed this mapping individually and then convened to discuss and compare results until an agreement was reached.

### Step 1: conduct a needs assessment

For this step, we integrated the findings of three previous studies (scoping review, Delphi study, collective case study) on TCIs and their implementation [3, 15, 31]. This task helped to determine the priority implementation factors (barriers, facilitators) that influence the implementation of different types of TCIs in practice. The Consolidated Framework for Implementation Research (CFIR) was applied to match the implementation factors to the relevant domains and constructs [35].

### Step 2: identify implementation outcomes, performance objectives, determinants, and change objectives

First, each of the implementation factors determined in step 1 was linked to its equivalent theoretical constructs (i.e., determinants) by considering the definition of each factor and understanding the essence or central meaning of the factor. We used the taxonomies of behavioral change and other relevant models or checklists to identify the equivalent theoretical constructs [23, 28, 36–38]. The core research team members performed this individually and then convened to discuss and compare results until an agreement was reached. Second, aided by the preliminary step on the description of the TCIs, we identified the implementation outcomes (referring to service and/or patient outcomes expected upon implementing a TCI), the actors (who will perform the actions needed to implement the core components of the TCIs), and the performance objectives (what do the actors have to do to promote the implementation of a TCI). Then matrices of change objectives were created, indicating what has to change in the determinants to bring about the performance objectives. AF developed the matrices and initially discussed these with researchers BdB and TvA, and following adjustments, then with the expert on Implementation Mapping, who advised on further alterations and enhancements.

### Step 3: select theoretical change methods and design implementation strategies

#### Step 3 (a)

In this step, theoretical change methods (strategies used to address the determinants relevant for each factor) at the individual or environmental level (including policy, social, and organizational) were selected from four taxonomies or overviews of change methods [20, 22, 23, 28, 30, 39]. These taxonomies or overviews indicate which strategies could be used to target the relevant determinants. Hence, a number of potential strategies were selected for each determinant. This step of mapping change methods to determinants was iterative whereby the core researchers performed it individually, after which they convened for four sessions to discuss and compare results until an agreement was reached.

#### Step 3 (b)

In this step, empirical evidence on the effectiveness of each of the strategies was assessed from published literature where possible. The search was guided mainly by considering systematic reviews and/or randomized-controlled trials or effectiveness-implementation hybrid design studies [40] conducted to demonstrate the effectiveness of strategies to implement innovations in either long-term care, transitional care, or general healthcare settings, as a first choice. In case only studies with other designs (qualitative, mixed-methods) were available to provide evidence on effectiveness, they were considered as a second choice. Moreover, when no evidence for a strategy was found in the literature, we referred to the relevant theory of change as a foundation for potential effectiveness. The main researcher (AF) performed the literature scan for evidence and then summarized the findings on the effectiveness of each strategy. Throughout this process, (AF) performed four individual consultation sessions with experts who provided feedback and advice on the strategies proposed (see description below) and the rationale for these. Furthermore, the three core team researchers discussed the available evidence on each strategy, which led to formulating a narrative conclusion on their effectiveness.

##### Expert consultation sessions -

Individual consultation sessions were performed with four scientific experts who have extensive knowledge and experience in the fields of implementation science, transitional care interventions, and long-term care. The experts were purposefully selected to cover all three areas of expertise and to make sure that the dominant area of expertise varied. Sessions were conducted online using a data-protected videoconferencing platform and each lasted an average of 1.5 h and was performed in the same manner and using the same content. The sessions aimed to discuss the various proposed implementation strategies; obtain feedback on their perceived importance, practicality, and applicability; and ask for further recommendations on sources of evidence on the effectiveness of, and suggestions for other strategies. This helped to iteratively refine the list of strategies.

#### Step 3 (c)

The core team held three iterative discussion sessions to determine the final selection of the core strategies based on (i) empirical evidence on effectiveness, (ii) support by the relevant theory of change, (iii) pragmatic rationale (feasibility, importance, practicality, and applicability of each strategy to the context of transitional care), and (iv) feedback from experts’ consultations. Consequently, practical applications (i.e., ways to apply and operationalize the strategy) were suggested for each selected implementation strategy, considering the context of transitional care. For each strategy, the target (i.e., who the strategy is directed at), and actor (i.e., who will deliver the strategy) were proposed [17].

## Results

We present the build-up of results for each step performed, leading to the final selection of implementation strategies for TCIs.

### Preliminary step — identification and description of the TCIs’ core components

Twenty different TCIs were identified from previous research work published elsewhere [3]. A total of 16 TCIs focused on improving care transitions while another four aimed to prevent transitions between care settings such as private homes, hospitals, intermediary care places, and nursing/residential care facilities. All 20 TCIs combined were found to encompass six out of the eight proposed core components of transitional care, as defined by Naylor and colleagues [34]. Table 2 describes the key elements of all 20 TCIs mapped to and combined under each of the six core components for transitional care (patient engagement, caregiver engagement, patient education, caregiver education, complexity management, and care continuity). Based on the existing TCIs, this table describes what a typical TCI is usually composed of and serves as a basic guide to key elements found in various innovations in transitional care. Care continuity presents as an extensive core component that prevails in the majority of TCIs. Hence, it is a backbone component specific to these innovations, given their nature to organize the continuum of care for older persons across different settings.

**Table 2:** Summary description of the TCIs’ elements as mapped to the six core components of transitional care

Core Component	Elements
**1. Patient Engagement**	▪ Establishment of trusting relationship with patient ▪ Development of rapport with patient, and understand the patient’s goals and preferences ▪ Active engagement of patient, family caregivers, and collaboration with primary care providers ▪ Active involvement of patients and informal caregivers for example in a triage decision-making for care transitions ▪ Integration of psychotherapeutic methods in care coordination and case management to increase patient engagement (e.g., motivational interviewing and behavioral activation)
**2. Caregiver Engagement**	▪ Establishment of trusting relationship with caregiver ▪ Active engagement of patient, family caregivers, and collaboration with primary care providers ▪ Active involvement of patients and informal caregivers for example in a triage decision for care transitions
**3. Patient Education**	▪ Discharge planning using “teach-back” methods ▪ Active engagement of patients and their family or informal caregivers by focusing on education and support ▪ Improvement of patient’s capacity in: medication self-management, using a patient-centered health record, knowledge of “red flags”, and making primary care provider/specialist appointments ▪ Provision of patient and caregiver education tools ▪ Coordination of education and community services to develop self-management skills ▪ Provision of a 30-day post-acute care bundle of transitional care services
**4. Caregiver Education**	▪ Building of the caregiver’s ability to identify early symptoms and apply strategies to prevent poor outcomes for patient ▪ Active engagement of patients and their family or informal caregivers by focusing on education and support ▪ Provision of patient and caregiver education tools
**5. Complexity Management**	▪ Development of individualized care plans, patient-caregiver goals (with patient, caregiver, and healthcare providers) ▪ Implementation of risk reduction strategies to minimize effects for example of cognitive impairment ▪ Daily hospital visits to patient-caregiver dyad as well as pre-discharge ▪ In-hospital patient case assessment and development of care plan ▪ Advanced care planning (assessment of needs at patient home and building a tailored care plan) ▪ Early identification and response to health risks of patient ▪ Comprehensive patient assessment within 3 days upon discharge by a home care nurse ▪ Development of a care plan based on input from patient and caregiver as well as a biopsychosocial needs assessment (post-discharge) ▪ Provision for example of a triage instrument for in-hospital assessment of patient needs for admission to a geriatric-rehabilitation unit before movement to a home setting ▪ Provision of acute-level care services at home as a substitute for hospital admission ▪ Identification, assessment, and management of acute conditions in a nursing home, such as evaluation, and communication of the resident status changes using communication tools (stop and watch warning tool), decision support (care pathways), and advance care planning ▪ Provision of a patient-centered holistic approach
**6. Care Continuity**^a^	*Majority of elements in this core component include a “Transition Role” with various tasks, described below* ▪ Presence of staff with a transition role such as *Advanced Practice Nurse* to perform: ✓ home/nursing facility visits 24 h post-discharge ✓ telephone follow-up and support ✓ coordination with a multidisciplinary local team of healthcare experts ▪ Presence of staff with a transition role such as *Transitional Care Manager* (social worker, or any other healthcare professional) to perform: ✓ discharge planning by management of environmental and community barriers ✓ coordination of transitional care (e.g., implementing home care, organizing home visits by professionals, delivering equipment to patient home, contacting long-term care placement agency, and reorienting patient when needed to rehabilitation services) ✓ exchange of patient information between providers ▪ Presence of staff with a transition role such as *Health Coach* (nurse or social worker) to perform: ✓ home visits (within 24–48 h) post-discharge ✓ follow-up phone calls and appointments with primary care providers ✓ connecting older adults to community services and resources ▪ Presence of staff with a transition role such as *Hospital Care Transition Nurse* to perform: ✓ patient care handoff between hospital care transition nurse and community rapid response nurse ✓ home care and follow-up period up to 30 days ✓ referral to hospital-based chronic disease management clinics ▪ Presence of staff with a transition role such as *Transitional Care Nurse* to perform: ✓ care coordination among providers and ensuring a multidisciplinary approach with open communication ✓ regular home visits and ongoing telephone support (7 days/week over 2 months post-discharge) ✓ continuity of medical care with hospital/primary care and accompanying patients on follow-up visits ▪ Presence of staff with transition role such as *Care Coordinator* to perform: ✓ home visits, telephone monitoring ✓ care coordination with a network of medical and social care providers in/out of hospital (including: symptoms management, functional management, psycho-support, medication management, promotion of self-care, referral to other services, appointment management, management of social issues, assessment of home environment) ▪ Presence of staff with transition role such as *Transition Coach* (nurse or social worker), to perform home visits post-discharge and follow-up telephone calls ▪ Presence of staff with transition role such as *Care Coordinator* (social worker) to perform: ✓ care coordination and follow-up in person or by telephone throughout 30 days post-discharge (e.g. provide brief counseling, arrange services and follow-ups, collaborate with other healthcare and social service providers) ▪ Presence of staff with transition role such as *Care Pathway Coordinator* in order to perform: ✓ coordination and continuity of care across settings ▪ Exchange of patient discharge information between the hospital, local healthcare allocations (municipality-level), and home care services, in order to: ✓ evaluate and decide on care assistance ✓ prepare home care service for transition ✓ provide general practitioner consults to patient 14 days post-discharge ✓ perform extended assessment during the first 4 weeks by district nurse/nursing assistant ▪ Delivery of transition care in temporary transition care places (e.g., low-intensity therapies and services, case management, and finalization of long-term care arrangements) ▪ Provision of community geriatric services (e.g., geriatrician and community nurse, 24 h telephone support and advisory service to nursing facility staff) ▪ Collaboration of hospital and community-based organizations for aging services (network collaboration) ▪ Availability of a Community Psychiatric Nurse, to perform follow-up visits and provide support/advice to family caregivers and facility nurses after the placement of an older person in a nursing home facility ▪ Provision of a 30-day post-acute period of transitional care bundle care coordination ▪ Combination of disease management in primary care settings and home care by coordinating care during an episode of acute illness across settings, and facilitated by a transitional care nurse

^a^Examples of key tasks given only, see reference Fakha et al. (2021) [3] for further details on multiple TCIs and key components of the transitional care roles they propose for delivering care continuity

### Step 1: conduct a needs assessment

Twelve factors were selected as key implementation factors for TCIs, based on the combined results of previous studies conducted by the research team [3, 15, 31]. These factors were reported and concluded as the most important to address in implementing TCIs. Table 3 describes these factors spanning the five domains of the CFIR. The majority of factors were at the organizational level (inner setting), such as the leadership commitment, involvement, and role in initiating the implementation of TCIs. Moreover, the availability of organizational resources along with the provision of access to knowledge and information on the TCIs were key factors to support the implementation. Continuous information exchange among various care providers involved in a care transition is another factor highlighted, as well as the sense of urgency to implement a TCI within an organization and the perception of it as a relative priority by individuals. Furthermore, one factor was linked to the outer setting and related to systems to finance the TCIs’ implementation. At the process level, engagement of key stakeholders and main participants in the implementation, as well as creating transition roles of staff, and evaluating the implementation process were regularly indicated to affect implementing TCIs. Furthermore, designing a TCI to match the care needs of the targeted groups (older persons) and considering the knowledge, beliefs, and personal attributes of healthcare professionals involved in implementing TCIs were also described as factors pertaining to each of the characteristics of the innovation and the individuals.

**Table 3:** Implementation factors with the corresponding equivalent determinants

Implementation Factor^a^ (description)	CFIR domain	Determinant (i.e., equivalent theoretical construct)
**Leadership** (Commitment, involvement, and accountability of leaders and managers with the implementation of a TCI. In addition, the presence of a skilled, motivated, and continuous leadership throughout the implementation, e.g., minimal turnover of dedicated project managers with high interest in the implementation.)	Inner setting^b^	▪ Commitment [28] ▪ Organizational commitment [36] ▪ Leadership [36]
**Engagement** (Attracting and involving appropriate individuals as below in the implementation and use of the intervention through a combined strategy of social marketing, education, role modeling, training, and other similar activities): 1. Key stakeholders (individuals from within the organization that are directly impacted by the TCI, e.g., staff responsible for making referrals to a new program or using a new work process), 2. Champions (individuals who dedicate themselves to supporting, marketing, and “driving through” an implementation, overcoming indifference or resistance that the intervention may provoke in an organization), 3. Innovation participants (individuals such as patients/older persons, family, and informal caregivers) served by the organization that participate in the TCI, (e.g. ensuring family inclusion in care goals setting) 4. Organizations/external context (developing and capitalizing on relationships with healthcare professionals and frontline staff in the implementing organizations, and promoting external collaborations with outside care providers (e.g. home care agency), and resources (e.g. community resources or social services for older persons) linked to the implementation of a TCI)	Process	▪ Attitudes [28] ▪ Beliefs [28] ▪ Motivation [36]
**Information continuity** (Care transitions' information continuity such as exchange of patient medical information, services, and care plans between healthcare providers. In addition, the continuity of steady work relationships between the healthcare providers and patients/caregivers and across all the organizations involved in the TCI implementation.)	Inner setting	▪ Environmental conditions (structural, organizational) [28] ▪ Social networks [28]
**Financing of TCIs’ implementation** (The existing financing structures that affect the TCI implementation such as fragmented financing and a lack of clear financing structures, or varied reimbursement systems of healthcare services.)	Outer setting	▪ Policy [28]
**Available resources and HIT systems** (The level of resources dedicated for the implementation and on-going operations of a TCI; including staffing levels, money, funding, training, education, physical space, equipment, and time. HIT – the electronic information management infrastructure and technologies, e.g., electronic health records available to clinicians to manage patient care, data, and communications.)	Inner setting	▪ Environmental conditions (structural, organizational) [28]
**Access to knowledge and information** (Ease of access to digestible information and knowledge, (e.g., mentoring, initial training) about the TCI and how to incorporate it into work tasks.)	Inner setting	▪ Environmental conditions (structural, organizational) [28]
**Sense of urgency** (The urgent need and attention given to implementing a specific TCI with respect to other innovation projects being addressed within an organization.)	Inner setting	▪ Risk perception [28] ▪ Organizational commitment [36]
**Relative priority** (Individuals’ e.g., healthcare professionals, staff within implementing team, shared perception of the importance of the implementation of a TCI within the organization)	Inner setting	▪ Attitudes [28] ▪ Beliefs [28] ▪ Organizational commitment [36]
**Reflecting and evaluating** (Quantitative and qualitative feedback about the progress and quality of implementation accompanied with regular personal and team debriefing about progress and experience.)	Process	▪ Feedback and reinforcement [28, 36] ▪ Monitoring [38]
**Targeted groups** (Patients/older persons who are the intended recipients or beneficiaries of the TCI, e.g., matching the care needs of older persons with high frailty or dementia.)	Intervention characteristics	▪ Innovation’s compatibility [37]
**Transition roles** (Administrative staff, providers within and outside the organization, e.g., frontline staff such as transition nurses or advanced practice nurses with designated transition roles who will carry out the TCI or be affected by it.)	Process	▪ Environmental conditions (structural, organizational) [28] ▪ Professional role [36]
**Knowledge, beliefs, and personal attributes of healthcare professionals** (Individuals’ attitudes toward and value placed on the intervention as well as familiarity with facts, truths, and principles related to the TCI, and other beliefs, expectations, and personal traits such as motivation levels, values, tolerance of ambiguity, critical attributes, intellectual ability, capacity, and learning style.)	Characteristics of individuals	▪ Knowledge [28] ▪ Attitudes [28] ▪ Beliefs [28] ▪ Motivation [36] ▪ Skills [28]

^a^Factors are listed in descending ranking order of priority, with leadership as most important; check reference for Delphi study [15] for further details

^b^Inner setting is also referred to as the organizational context

### Step 2: identify implementation outcomes, performance objectives, determinants, and change objectives

Table 3 lists the multiple determinants (i.e., relevant theoretical constructs) identified for each of the 12 factors (e.g., attitudes, beliefs, and motivation for the factor engagement). In addition, based on the preliminary step, we determined the relevant TCIs’ implementation outcomes, the corresponding necessary performance objectives to achieve these outcomes, and the actors: (i) leaders and organizations and (ii) healthcare professionals. Determinants were allocated to the pertaining performance objectives. Hence, two matrices of change were developed for each type of actor, and we formulated the change objectives linked to the performance objectives and determinants. The matrices of change are presented in Additional file 2. As an example, one performance objective was to engage the patient and caregiver, which is linked to the determinants (attitudes and beliefs of healthcare professionals), and hence the corresponding change objectives were to believe that the TCI is beneficial to enhance transitional care for older persons and to express a positive attitude towards the TCI as an innovation.

### Step 3: select theoretical change methods and design implementation strategies

#### Step 3 (a, b)

This step was completed by selecting strategies (i.e., theoretical change methods) expected to address the determinants and change objectives identified in step 2. An extensive list of strategies, such as modeling and active learning from the Social Cognitive Theory, consciousness raising from the Trans-Theoretical Model, persuasive communication from the Communication-Persuasion Matrix, and belief selection from the Theory of Planned Behavior, was identified [23, 28]. Theories of Organizational Development and Organizational Readiness for Change provided a number of strategies to address determinants such as structural influences and organizational commitment [23, 28, 41]. Other selected strategies included building a coalition, conducting local consensus discussion, role expansion/task shifting, and revising professional roles [20, 30]. All identified methods focused on either the organizational level (e.g., home care organization and hospital) or at the individual level (e.g., general practitioners and nursing home staff) (see Table 4). For example, persuasive communication can be used to create convincing arguments on the importance and effectiveness of a TCI in improving care transitions and hence address the commitment (determinant) of leadership to implement a new TCI in an organization. Similarly, to improve the knowledge and attitudes of healthcare professionals, using guided practice as a method to help train their ability to deliver the components of a TCI can address the skills (determinant) of the professionals involved in implementing the TCI.

**Table 4:** List of selected implementation strategies, description, and relevant theory and/or evidence on effectiveness

**Factor**	**Strategy (change method)**	**Description**	**Relevant theory and/or evidence from literature**^a^
**Leadership**	**1. Persuasive communication**	Guiding individuals and environmental agents toward the adoption of an idea, attitude, or action by using arguments or other means	*Communication-Persuasion Matrix, Elaboration Likelihood Model, Diffusion of Innovations Theory* [23, 28] ➣ The use of persuasive, novel, relatable, and important arguments and communication with leaders can be effective to achieve change
	**2. Public commitment**	Stimulating pledging, promising or engaging oneself to perform the healthful behavior, and announcing that decision to others	*Theories of Automatic, Impulsive and Habitual Behavior* [23, 28] ➣ **Indications for positive effect:** organizational leaders committed to implementing an intervention to reduce hospitalization from a nursing home directly increased the staff commitment to implementation [42]
	**3. Sense-making**	Leaders reinterpret and relabel processes in organization, create meaning through dialogue, and model and redirect change	*Organizational Development Theory* [23, 28] ➣ **Indications for positive effect:** a committed organizational leadership who created an encouraging environment within healthcare organizations (e.g., supportive policies and practices, allocation of funds and resources) to implement an innovation increased directly the staff commitment to implementation [43]
	**4. Development of leadership skills**	Development of strong leadership skills in clinical leaders to align and sustain evidence-based practice implementation efforts	➣ **Indications for positive effect:** demonstrated as useful to build leadership skills to support the implementation of evidence-based practices in community-based mental health organizations [44]
**Engagement**	**5. Participation**	Assuring high level engagement of the participants’ group in problem solving, decision making, and change activities; with highest level being control by the participants’ group	*Diffusion of Innovations Theory, Theories of Power, Organizational Development Theories, Models of Community Organization* [23, 28] ➣ **Indications for positive effect:** engaging stakeholders (e.g., managers, local providers) through meetings, briefings, and consultations increased the reach and adoption of a mental health intervention in primary care clinics [45] ➣ Patient activation improved health-related outcomes suggesting substantial positive effects [46]
	**6. Modeling**	Providing an appropriate model being reinforced for the desired action	*Social Cognitive Theory* [47] ➣ Providing models (similar persons) that can do the same tasks and demonstrate steps to attain a complex objective can be effective to motivate and increase self-efficacy ➣ **Indications for positive association:** *champions* were related to increased use of innovations in healthcare organizations [48] / identifying *early adopters* and learning from their experiences was associated with the uptake of a new evidence-based treatment in a clinical care setting [49, 50] ➣ **Indications for positive effect:** *opinion leaders* can probably improve healthcare professionals' compliance with evidence-based practice (effect sizes are modest and varied) [51]
	**7. Build a coalition**	Recruit and cultivate relationships with partners in the implementation effort	➣ **Indications for positive association:** building local teams to address challenges were associated with the uptake of a new evidence-based treatment in a clinical care setting [49, 50]
	**8. Conduct local consensus discussions**	Include local providers and other stakeholders in discussions that address whether the chosen problem is important and whether the clinical innovation to address it is appropriate	➣ **Indications for positive effect:** local consensus discussions proved effective to improve adherence to a new guideline in a nursing home (used in combination with other strategies) [52]
	**9. Use mass media**	Use media to reach large numbers of people to spread the word about the clinical innovation	➣ **Indications for positive effect:** evidence showed that these channels of communication may have an important role in influencing the use of healthcare interventions and services [53]
**Information continuity**	**10. Structural redesign**	Change organizational elements such as formal statements of organizational philosophy, communication flow, reward systems, job descriptions, and lines of authority	*Organizational Development Theory* [23, 28] ➣ Planned development, improvement, or reinforcement of structures and processes can promote organizational effectiveness and help deal with resistance to change
	**11. Change in communication between providers, including distant ones**	Systems to improve the communication between healthcare providers, including establishment of any type of telecommunication link for implementation (e.g., use of information and communication technology)	➣ **Indications for positive effect:** the use of HIT communication systems improved adherence to a new guideline in a nursing home (used in combination with other strategies) [54]
	**12. Enhancing network linkages**	Training network members to provide support and members of the target group to mobilize and maintain their networks	*Theories of Social Networks and Social Support* [23, 28] ➣ **Indications for positive association:** promoting working relationships and networks inside/outside the organization was associated with the uptake of a new evidence-based treatment in a clinical care setting [49, 50]
**Financing of TCIs’ implementation**	**13. Advocacy and Lobbying**	Arguing and mobilizing resources on behalf of a particular change, giving aid to a cause, active support for a cause or position	*Stage Theory of Organizational Change; Models of Community Organization; Agenda-Building Theory; Multiple Streams Theory* [23, 28] ➣ Is a primary method to achieve change at the environmental/community level and highlights the role of community activism to support important issues
**Available resources and HIT systems**	**14. Changes in Staffing models**	Interventions to achieve an appropriate level and mix of staff, recruitment and retention of staff, and transitioning of healthcare workers from one environment to another, for example interventions to increase the proportion of healthcare workers in underserved areas	➣ **Indications for positive association:** forming new interdisciplinary teams was associated with the uptake of a new evidence-based treatment in a clinical care setting [49, 50]
	**15. Use of HIT systems**	Health record and health management systems to store and manage patient health information, for example electronic patient records, or systems for recalling patients for follow-up or prevention	➣ **Indications for positive effect:** the use of HIT communication systems improved adherence to a new guideline in a nursing home (used in combination with other strategies) [54]
	**16. Develop resource sharing agreements**	Develop partnerships with organizations that have resources needed to implement the innovation	*Organizational theories – Resource dependency theory* [55] ➣ **Indications for positive association:** partnering with other resourceful organizations can help implement changes and was associated with the uptake of a new evidence-based treatment in a clinical care setting [49, 50]
**Access to knowledge and information**	**17. Facilitation**	Creating an environment that makes the action easier or reduces barriers to action	*Social Cognitive Theory* [47] ➣ **Indications for positive effect:** improved the adoption of evidence-based guidelines in various clinical areas that focused on prevention, system-level improvements, and outcomes associated with chronic disease management within clinical practice settings [56]
	**18. Develop and distribute educational materials**	Develop guidebooks, manuals, toolkits, and other supporting materials in ways that make it easier for stakeholders to learn about the innovation and for clinicians to learn how to deliver the clinical innovation, and distribute it in person, by mail, and/or electronically	➣ **Indications for positive effect:** can probably improve healthcare professionals' practice but slightly, and electronic versions have little or no difference compared to printed versions (effect sizes are small and variable) [57]
	**19. Conduct educational meetings and ongoing trainings**	Hold meetings and trainings (e.g., courses, workshops, conferences) in an ongoing way targeted toward different stakeholder groups (e.g., providers, administrators, other organizational stakeholders, and community, patient/consumer, and family stakeholders) to teach them about the clinical innovation	➣ **Indications for positive effect:** can probably improve compliance with desired practice, and interactive formats are more effective than didactic ones (effect sizes are modest) [58]
	**20. Create a learning collaborative**	Facilitate the formation of groups of providers or provider organizations and foster a collaborative learning environment to improve implementation of the clinical innovation	➣ **Indications for positive association:** improvement in the adoption and implementation quality of expanding new clinical services in primary care settings were moderately associated with the engagement of clinicians in learning collaboratives [59]
**Sense of urgency**	**21. Consciousness raising** **&**	Providing information, feedback, or confrontation about the causes, consequences, and alternatives for a problem or a problem behavior	*Health Belief Model, Precaution-Adoption Process Model, Trans-Theoretical Model* [23, 28] ➣ Providing cues for action or information about risk is important and can be effective to change perceptions and implement change
	**22. Scenario-based risk information**	Providing information that may aid the construction of an image of the ways in which a future loss or accident might occur	
	**23. Organizational diagnosis and feedback**	Assessing of organizational structures and employees’ beliefs and attitudes, desired outcomes and readiness to take action, using surveys and other methods	*Organizational Development Theory, Organizational Readiness for Change* [23, 28, 41] ➣ Assessing an organization's readiness and accordingly fostering its capacity, capabilities, commitment, and efficacy to change are key drivers to developing readiness to implement change within an organization
**Relative priority**	**24. Belief selection**	Using messages designed to strengthen positive beliefs, weaken negative beliefs, and introduce new beliefs	*Theory of Planned Behavior, Reasoned Action Approach* [23, 28] ➣ Identifying the beliefs and awareness level of the receiver regarding the problem and its negative outcomes, then providing a relevant positive evaluation for a recommended behavior can be effective to achieve change
	**25. Persuasive communication**	Guiding individuals and environmental agents toward the adoption of an idea, attitude, or action by using arguments or other means	*Communication-Persuasion Matrix, Elaboration Likelihood Model, Diffusion of Innovations Theory* [23, 28] ➣ The use of persuasive, novel, relatable, and important arguments and communication with leaders can be effective to achieve change
	**26. Organizational diagnosis and feedback**	Assessing of organizational structures and employees’ beliefs and attitudes, desired outcomes and readiness to take action, using surveys and other methods	*Organizational Development Theory, Organizational Readiness for Change* [23, 28, 41] ➣ Assessing an organization's readiness and accordingly fostering its capacity, capabilities, commitment, and efficacy to change are key drivers to developing readiness to implement change within an organization
**Reflecting and evaluating**	**27. Audit and feedback**	A summary of health workers’ performance over a specified period of time, given to them in a written, electronic or verbal format. The summary may include recommendations for clinical action	*Theories of Learning, Goal-Setting Theory, Social Cognitive Theory* [23, 28] ➣ **Indications for positive effect:** can probably lead to important improvements in professional practice (effect sizes are small), yet is most effective when the performance is low, provided regularly by supervisors or colleagues, and includes clear targets/action plans [60]
	**28. Monitoring the performance of the delivery of healthcare**	Monitoring of health services by individuals or healthcare organizations, for example by comparing with an external standard	➣ **Indications for positive effect:** improved adherence to a new guideline in a nursing home (used in combination with other strategies) [52, 54]
**Targeted groups**	**29. Tailoring**	Matching the intervention or components to previously measured characteristics of the participant	*Trans-Theoretical Model, Precaution Adoption Process Model, Protection Motivation Theory, Communication-Persuasion Matrix* [23, 28] ➣ **Indications for positive effect:** suggested as important and essential to design evidence-based treatments in mental health [61] / can probably lead to the successful implementation of innovations in long-term residential care [62]
**Transition roles**	**30.Structural redesign**	Change organizational elements such as formal statements of organizational philosophy, communication flow, reward systems, job descriptions, and lines of authority	*Organizational Development Theory* [23, 28] ➣ Planned development, improvement, or reinforcement of structures and processes can promote organizational effectiveness and help deal with resistance to change
	**31. Role expansion or task shifting** **&**	Expanding tasks undertaken by a cadre of health workers or shifting tasks from one cadre to another, to include tasks not previously part of their scope of practice	➣ **Indications for positive effect:** showed improvements in the process of care outcomes [56] / was associated with the uptake of a new evidence-based treatment in a clinical care setting [49, 50]
	**32. Revise professional roles**	Shift and revise roles among professionals who provide care, and redesign job characteristics	
**Knowledge, beliefs, and personal attributes of healthcare professionals**	**33. Active learning**	Encouraging learning from goal-driven and activity-based experience	*Elaboration Likelihood Model, Social Cognitive Theory, Theories of Information Processing, Theories of Stigma and Discrimination, Communication-Persuasion Matrix, Theories of Learning, Theory of Planned Behavior, Reasoned Action Approach* [23, 28] ➣ All these methods can help improve the individual’s capabilities and aptitude to implement change
	**34. Chunking**	Using stimulus patterns that may be made up of parts but that one perceives as a whole	
	**35. Advance organizers**	Presenting an overview of the material that enables a learner to activate relevant schemas so that new material can be associated	
	**36. Shifting perspective**	Encouraging taking the perspective of the other	
	**37. Arguments**	Using a set of one or more meaningful premises and a conclusion	
	**38. Direct experience**	Encouraging a process whereby knowledge is created through the interpretation of experience	
	**39. Belief selection**	Using messages designed to strengthen positive beliefs, weaken negative beliefs, and introduce new beliefs	
	**40. Guided practice**	Prompting individuals to rehearse and repeat the behavior various times, discuss the experience, and provide feedback	*Social Cognitive Theory; Theories of Self-Regulation* [23, 28] ➣ **Indications for positive effect:** coaching can lead to the implementation of more quality improvement interventions for cardiovascular health [63]

Health information technology (HIT), ^a^Empirical evidence and relative explanation from literature are provided where available, Positive association indicates that a correlation is likely present between the strategy and a successful implementation of change/innovation; Positive effect indicates that a causal relationship is likely present between the strategy and a successful implementation of change/innovation, (&) where present indicates that the two strategies share the same references/articles on evidence of effects, the term change method is equivalent to implementation strategy that is used to address the determinant relevant for each factor

The list of identified strategies was iteratively refined following feedback from the experts’ consultation sessions, who reviewed, validated, and proposed new strategies or amendments. Specifically, the experts confirmed the selection of strategies to address the leadership skills and capabilities, as they were considered a priority for implementing TCIs. Moreover, they emphasized the importance of the strategy “participation,” and the inclusion of certain aspects within it such as stakeholder mapping and building an interdisciplinary coalition across care settings. Similarly, strategies to enhance communication and information exchange, and networking across care settings and healthcare professionals were proposed as essential for implementing TCIs. The strategy of “advocacy and lobbying” at the policy level was indicated by experts as difficult and time-consuming, yet important to keep in order to have continuous catalysts to lobby for implementing innovations in transitional care. Tailoring the TCIs was also considered a necessary strategy by the experts and was proposed to be operationalized as conducting local care and needs assessment to make the innovation context/target population specific.

A number of strategies were supported by either only theory (e.g., consciousness raising) or only evidence from the literature (e.g., conduct local consensus discussions), while others were supported by both (e.g., modeling, participation). Evidence on the effectiveness of some identified strategies to achieve change was retrieved from the literature and where possible strategies could be denoted as having either a positive effect or association to implement an innovation in a care setting. Moreover, some strategies were indicated as having various degrees of effects such as small, modest, and moderate. Many studies explored the effects of a combination of strategies (multifaceted) on implementing change [49, 50, 59, 63], and few were specific to long-term care settings and transitional care [42, 52, 54]. Table 4 describes the summary list of the final implementation strategies that were selected and details the relevant theory and/or evidence on effectiveness for each.

#### Step 3 (c)

Eventually, a total of 40 strategies were selected, four of which (persuasive communication, belief selection, structural redesign, and organizational diagnosis and feedback) address more than one factor (see Table 4). The majority of strategies (*n* = 21) were at the organizational level and almost half were supported by evidence as having a positive effect on implementing change such as TCIs in practice [42, 43, 52, 54, 56]. For example, facilitation and creating a supportive organizational environment proved as effective to improve the adoption and implementation of new guidelines in clinical settings [56]. Likewise, using communication systems including health information technology (HIT) to improve information continuity among providers within and across care organizations improved adherence to new guidelines in a nursing home [54]. Sense-making was another strategy that can effectively address organizational leadership and foster leaders’ commitment to implementing an innovation such as TCIs [43]. Some strategies (e.g., building a coalition, enhancing network linkages, changes in staffing models, and developing resource sharing agreements) exhibited a positive — but not necessarily causal — association with implementing change, and they were selected due to their high relevance to the context of transitional care, whereby multiple care settings and organizations are usually involved in implementing TCIs [49, 50, 59]. Other selected strategies (e.g., audit and feedback, educational materials, educational meetings/training) at the organizational level are commonly used and presented with effect sizes on implementing change, hence they were considered essential in the implementation of TCIs [57, 58, 60].

Strategies at the individual level (*n* = 13) such as belief selection and scenario-based risk information, were all supported by theory. Participation, modeling, and guided practice were the only three strategies that were supported by evidence, in addition to theory, as having a positive effect or association [45, 46, 48–51, 63]. For example, engaging all the key stakeholders in an implementation effort early on and continuously could directly improve the adoption and implementation of an innovation.

Otherwise, one strategy at the policy level (i.e., advocacy and lobbying) was selected, and another at the innovation level (i.e., tailoring). Evidence on tailoring as a strategy indicated a positive effect, hence matching the TCIs’ components to the care needs of older persons is considered essential for successful implementation [61, 62].

Afterward, suggestions on how to operationalize the selected strategies were created as practical applications, and the corresponding target was proposed (see Table 5 for a full description). For example, a public announcement of the introduction of a new TCI made by an organizational leader and included in a newsletter could increase the organizational commitment to implementing the TCI.

**Table 5:** List of selected implementation strategies, suggested practical applications, and target

Factor	Strategy (change method)	Practical applications (suggested)	Target (directed at)
**Leadership**	**1. Persuasive communication**	▪ Organize meetings with organizational leaders to introduce the new TCI and deliver convincing arguments and important messages (e.g., evidence on the effects of TCIs, need for person-centeredness, etc.) ▪ Perform open discussions with organizational leaders to discuss objections, change their attitude/perspective, and guide them toward adopting the TCI	*Leaders of organizations (including management staff)*
	**2. Public commitment**	▪ Obtain formal written commitments from organizational leaders to implement the TCI ▪ Make a public declaration/announcement expressing organizational leaders’ determination to implement the TCI (e.g., put proof on websites, or in a newsletter)	*Leaders of organizations (including management staff)*
	**3. Sense-making**	▪ Assess and analyze current care integration and care continuity practices, then reinterpret to signal shortcomings and the need to provide transitional care in a new or different way ▪ Recreate processes and redefine organizational ethos/culture to support innovations and new ideas for transitional care	*Organizational board of directors, executives, Quality improvement department*
	**4. Development of leadership skills**	▪ Identify formal and informal leaders and assess the need for leadership skills-building ▪ Train on the change effort (e.g., group-based interactive and didactic training sessions, following the leadership and organizational change for implementation LOCI training) ▪ Provide coaching on the job by an external expert to promote the leaders' readiness and skills	*Leaders of organizations (including management staff)*
**Engagement**	**5. Participation**	▪ Identify relevant key persons (stakeholders) ▪ Map stakeholders by their influence, power, authority, and importance in relation to implementing the TCI ▪ Activate stakeholder groups (e.g. older persons associations, long-term care lobbyists) ▪ Identify issues in suboptimal transitional care and involve stakeholders to take control and be accountable to resolve the problems jointly	*Stakeholders*
	**6. Modeling**	▪ Identify role models within the local context (e.g., champions, early adopters, opinion leaders) ▪ Support the role models to lead the TCI and to help influence and motivate fellow colleagues to adopt the TCI and help drive the implementation ▪ Invite the role models to share their experiences with the TCI in practice (e.g., testimonials, informal meetings to share stories)	*Healthcare professionals*
	**7. Build a coalition**	▪ Organize meetings and information sessions across different care settings (e.g., hospital, homecare, social care) to increase mutual understanding about the TCI implementation workflow, i.e., align on common goals, values, and needs	*Organizations, Healthcare professionals*
	**8. Conduct local consensus discussions**	▪ Conduct discussions with staff of different care settings (e.g., hospital, homecare, social care) to agree on core problems, needs, and goals, and to assess the benefit of implementing the TCI to address issues in delivering transitional care	*Organizations, Stakeholders*
	**9. Use mass media**	▪ Spread information about the TCI and its benefits using media channels (e.g., a quick guide on the website, refresher sessions, newsletters, and social media)	*Stakeholders*
**Information continuity**	**10. Structural redesign**	▪ Develop a formal communication workflow, and a guide for healthcare professionals to integrate care collaboratively and to share information between different care settings (e.g., hospital, homecare, social care) involved ▪ Develop a standardized discharge letter to indicate the treatment plan upon transition from one care setting to another (e.g., hospital to home care)	*Organizations, Healthcare professionals*
	**11. Change in communication between providers, including distant ones**	▪ Develop HIT systems interlinked between different organizations, or find ways to automatize information exchange between systems when possible ▪ Optimize communication links (e.g., smartphone communication apps, conferencing technology, web chat, email)	*Organizations*
	**12. Enhancing network linkages**	▪ Create inter-organizational networks (common platforms, regular conferences and meetings, collaborative learning sessions) to discuss issues and updates in transitional care delivery ▪ Conduct teams meetings for staff implementing the TCI to share experiences, problem-solving, and support one another	*Organizations, Healthcare professionals*
**Financing of TCIs’ implementation**	**13. Advocacy and Lobbying**	▪ Develop a proposal for new funding formulas supportive of healthcare providers to implement innovations in transitional care ▪ Draft a new policy brief and business case supportive of improving transitional care ▪ Identify key funders in the system and request discussions with them ▪ Identify the key TCIs’ leaders and prepare the advocacy and lobbying with them	*National healthcare system, Ministry of health, Regional public health governance, Health insurers, Funding agencies*
**Available resources and HIT systems**	**14. Changes in Staffing models**	▪ Develop a proposal for new staffing arrangements (number, roles) ▪ Develop a proposal for retaining and attracting transitional care experts (e.g., care transition nurse)	*Organizations, Policymakers, Funders*
	**15. Use of HIT systems**	▪ Assess existing/non-existing HIT systems ▪ Develop a proposal to use and potentially integrate HIT systems to share medical information in care transitions and to support TCI implementation	*Organizations*
	**16. Develop resource sharing agreements**	▪ Establish formal agreements (e.g., memorandum of understanding) between LTC organizations on resource sharing ▪ Create formal/informal networks between LTC organizations	*Organizations*
**Access to knowledge and information**	**17. Facilitation**	▪ Use change agents/facilitators to build a relationship with healthcare professionals ▪ Provide practical support, relevant materials and means, and information to enhance the use of TCIs (e.g., helpdesk, outreach support, information zone)	*Healthcare professionals*
	**18. Develop and distribute educational materials**	▪ Develop supporting educational materials (e.g., manual, toolkit, brochures, guidebook, reminders, videos) to inform about the TCI and how to implement it, including technology-delivered content (e.g., online, smartphone applications)	
	**19. Conduct educational meetings and ongoing training**	▪ Conduct training, workshops, conferences, lectures, and meetings to inform about the TCI and how to implement it (use both didactic and interactive formats)	
	**20. Create a learning collaborative**	▪ Foster the formation of a long-term network group of healthcare professionals for learning and exchanging ideas on how to provide optimal transitional care ▪ Organize educational outreach visits by a trained person to meet with healthcare professionals in their work settings to educate them about the TCI and provide support in using it ▪ Establish academic partnerships with a university or academic unit to share research skills on implementing the TCI ▪ Use video stories from other organizations that implemented a TCI	
**Sense of urgency**	**21. Consciousness raising &** **22. Scenario-based risk information**	▪ Use media to spread testimonials of what went wrong during care transitions ▪ Conduct informational meetings to raise awareness and highlight the need for the TCI (use a real case to discuss risks and adverse outcomes of poor care transitions) ▪ Provide stories or videos depicting alternative courses of events as told by care staff healthcare workers can identify with	*Leaders of organizations (including management staff), Healthcare professionals*
	**23. Organizational diagnosis and feedback**	▪ Assess the various organizational aspects that should be in place and the organization’s degree of readiness to implement the TCI (e.g., use surveys, and focus groups) ▪ Establish a list of barriers and facilitators in the organization that can influence the implementation of the TCI and how to overcome/leverage them ▪ Enter into discussions with organizational leaders on the above and explore solutions	*Organizations*
**Relative priority**	**24. Belief selection &** **25. Persuasive communication**	▪ Provide information and testimonials about the positive/negative consequences of implementing/not implementing the TCI (e.g., cases, stories) ▪ Share findings of needs assessment (e.g., case report) and describe the priority to implement the TCI within the organization	*Leaders of organizations (including management staff), Healthcare professionals*
	**26. Organizational diagnosis and feedback**	▪ Conduct a survey to collect perspectives on implementing the TCI ▪ Discuss the results with organizational and operational leaders and explore solutions with them	*Organizations*
**Reflecting and evaluating**	**27. Audit and feedback**	▪ Prepare and perform audits for assessing the quality of transitional care ▪ Develop a summary of performance regarding the TCI implementation (key measures on process /outcomes) over a specific period ▪ Organize regular implementation team meetings to relay data on performance (provide feedback), reflect on implementation effort (get feedback), and share lessons learned	*Organizations, Healthcare professionals*
	**28. Monitoring the performance of the delivery of healthcare**	▪ Develop tools/systems for quality monitoring with measures specific to the TCI implemented (e.g., processes, patient outcomes, implementation outcomes) ▪ Assess continuously the progress of the TCI implementation and adjust clinical practices when needed	
**Targeted groups**	**29. Tailoring**	▪ Asses the local care and social needs of the target population of older persons ▪ Develop an adaptable TCI (clarify the core and modular components) to meet the local needs	*Older persons*
**Transition roles**	**30. Structural redesign,** **31. Role expansion or task shifting, &** **32. Revise professional roles**	▪ Map current inter-organizational interactions, work processes, and staff roles and identify changes necessary to accommodate the TCI implementation ▪ Expand, shift, or revise job descriptions, roles, and tasks of staff to include implementing the TCI	*Organizations, Healthcare professionals*
**Knowledge, beliefs, and personal attributes of healthcare professionals**	**33. Active learning**	▪ Conduct interactive lectures, group discussions, and teach-back sessions (e.g., exercises with a workbook, case study solving) about the TCI components and features	*Healthcare professionals*
	**34. Chunking &** **35. Advance organizers**	▪ Use labels, color-coding, index in the educational materials about the TCI ▪ Structure and write texts in the most accessible way	
	**36. Shifting perspective,** **37. Arguments, &** **38. Belief selection**	▪ Challenge organizations and healthcare professionals to take the perspective of an older person facing a care transition and ask them to consider what they would find important ▪ Organize meetings with healthcare professionals to introduce the new TCI and deliver convincing arguments and important messages (e.g., evidence on the effects of TCIs, need for person-centeredness, etc.) ▪ Perform open discussions with healthcare professionals to discuss objections, and ways to overcome them, and provide guidance towards adopting the TCI (e.g., integrate new beliefs about the TCI)	
	**39. Direct experience** **40. Guided practice**	▪ Assign to a care setting where a TCI is implemented to obtain direct observation ▪ Bring experts on innovations in transitional care to model tasks and skills required and to provide ongoing implementation support on-site ▪ Bring TCI coaches to support on innovations, transitional care, and implementation	*Healthcare professionals*

All strategies to be performed by the *Actor —* individual/entity that will deliver the implementation strategy and wants to implement a TCI and improve care transitions in long-term care (e.g., administrators, payer, provider, leader, manager, researcher, implementation specialist); *Practical applications:* are suggestions on how to operationalize the strategies (i.e., implementation activities), and they can be adapted and further expanded as needed; *Target:* individual/entity that will decide to uptake a TCI and put it in practice (e.g., transitional care nurse); *Organizations:* all long-term care organizations involved in improving care transitions between settings and implementing a TCI; *Stakeholders:* important individuals who are key and have a great influence on the implementation success, includes healthcare providers, direct implementation staff, leaders, patients/older persons, informal caregivers, family; *Healthcare professionals:* frontline staff who are directly involved in implementing the TCI; *TCI*, transitional care innovation; *LTC*, long-term care; the term change method is equivalent to implementation strategy that is used to address the determinant relevant for each factor

## Discussion

Despite the rapidly increasing development and implementation of various innovations in transitional care and healthcare in general, literature highlighted common challenges related to selecting and using implementation strategies [16]. Limited assessment of implementation factors, insufficient use of a systematic method to develop implementation strategies, and little consideration of relevant theories and evidence in the selection of strategies are key issues that impede the success of implementing innovations, such as TCIs [16, 32, 64]. The current project is a novel work that applied Implementation Mapping [32] and developed a set of implementation strategies carefully selected for TCIs. Initially, our findings identified 20 TCIs whereby the majority aimed to improve care transitions and had care continuity including the presence of staff with transition roles as a fundamental component. Consequently, we determined 12 priority factors, mainly linked to the organizational setting, which influence the implementation of TCIs and hence require specific strategies to address them. This culminated in the formulation of a set of various implementation strategies at the organizational, individual, policy, and innovation levels. We systematically selected strategies supported by theory and evidence on their effectiveness in implementing change in healthcare settings. The larger part of the selected implementation strategies aimed at targeting the organizational commitment for change, leadership behaviors and skills, and structural features. In addition, key strategies were selected to enhance the individuals’ attitudes, awareness, beliefs, knowledge, and skills to implement TCIs. Fewer strategies were selected at the policy and innovation levels, which could be explained by less implementation factors reported at these levels.

The final selection of implementation strategies is more comprehensive than earlier projects that developed strategies to implement TCIs and which focused only on one TCI and did not follow a thorough procedure such as Implementation Mapping [10, 26]. However, similar to our selection, the majority of the strategies used in these projects were also at the organizational level (i.e., audit and feedback, revise professional roles) [10]; whereas strategies used in projects to implement for example health promotion interventions tend to be more at the individual level [65, 66]. Furthermore, our selection of strategies corresponds to and expands further on the list of strategies proposed by McArthur et al., designed to improve the implementation of evidence-based guidelines in long-term care and which included education, training, environmental restructuring, persuasion, modeling, and enablement [67].

As for the large number of selected strategies, we opted to propose multiple potential strategies for each factor given the heterogeneity of organizations (e.g., hospital, homecare, nursing home, and transition unit) involved in transitional care. The variety of strategies to choose from allows for further tailoring to the different settings where TCIs may be implemented.

Further, the selected implementation strategies align with the guidelines on describing and operationalizing strategies recommended by Proctor et al. [17]. Though, while we suggested potential actors (i.e., individuals and entities to deliver the strategies), we did not specify the dose and temporality for each strategy. Hence, the dosage (e.g., number of training sessions, frequency of monitoring, or amount of time spent with an expert on implementing innovations) of each strategy should be considered by actors and possibly set beforehand to achieve a certain effect. Likewise, temporality or sequence of strategy use is critical and should be well thought of, e.g. assessing the organizational aspects needed for implementing a TCI should precede any structural redesign or changes in staffing models.

Literature on the empirical evidence on the effectiveness of the strategies was limited. Most studies in general healthcare concentrated on examining the effects of a few strategies such as audit and feedback, educational materials and activities, clinician reminders, opinion leaders, and revising professional roles [27, 56, 68]. Moreover, evidence for strategies used to implement innovations specifically in the context of transitional care or long-term care was scarce. It is also important to note that many studies evaluated the effects of multiple implementation strategies used together simultaneously rather than individually, which thus makes it harder to disentangle the relative effect per strategy. Furthermore, there was a lack of clear, consistent, and detailed descriptions of the strategies used in various empirical studies. This resonates with known constraints to interpret implementation efforts, which hinders the ability to comprehend the success and effectiveness of implementation strategies [69].

### Implications for practice and research

We emphasize again that the use of implementation strategies is a focal task in the prospective implementation of any TCI in practice. Hereby, we suggest to future implementers to first perform an analysis of the setting at hand, and to consider the priority factors we identified as most likely present in their respective context. Second, to utilize the relevant implementation strategies resulting from this project as both a backbone and starting point for implementing a TCI. Further deliberations among implementers of a specific TCI are also recommended and may be necessary to choose from the strategies which are feasible, applicable, and perceived as important in relevance to their particular context.

Moreover, it is imperative to indicate that the selected implementation strategies are neither country-specific, nor particular to one healthcare system (e.g., USA healthcare system or Dutch healthcare system). The strategies are rather generic and their relative importance might vary across every context (e.g., country, healthcare setting), and hence further tailoring is needed when selected for use in a certain context (i.e., healthcare legislation, healthcare financing system).

This work merits further research and notably empirical studies to assess the effectiveness of the implementation strategies in the context of transitional care. Other suggested investigations may include assessing the use of different combinations of strategies as well as if a potential hierarchy of importance or optimal sequencing can maximize effects. This will help add to the currently limited evidence on implementation strategies.

### Strengths and limitations

To our knowledge, this project is the first to apply Implementation Mapping as recommended by Fernandez et al. [32] to improve specifically the implementation of TCIs in long-term care settings. Therefore, our approach was valuable to advance efforts to better implement innovations in transitional care. This method allowed us to identify and understand factors thoroughly and then to use theory and evidence to deliberately select strategies with specific mechanisms adept to bring about the desired change in the behaviors of individuals or organizations [70]. Furthermore, we performed deliberate preparatory work for this project beforehand by conducting a series of research studies on the implementation of TCIs. Lastly, we acknowledge that the selected strategies can be similar to strategies used for implementing other types of innovations in long-term care. Yet, our strategies thoroughly selected to address factors that influence specifically the implementation of TCIs can be more effective than strategies not specially selected for the implementation of TCIs [71].

On the other hand, we note some limitations of this project. First, the views and interpretations of the research team might have affected certain steps of the Implementation Mapping process. This includes the final consideration and judgment of whether to select a strategy or not. However, we tried to limit subjectivity by obtaining different perspectives from experts in the fields of implementation science and transitional care. Moreover, involving more practitioners and direct implementers of TCIs could have been worthwhile to this work. Second, we performed a rapid scan of empirical evidence for the strategies rather than a systematic literature search. Therefore we might have missed some studies on evidence for certain strategies, yet we used a flexible terminology/description for strategies in our search and explored multiple databases. However, it is important to highlight that we experienced a literature gap in empirical evidence for implementation strategies, which might have influenced our final selection of strategies.

## Conclusions

The implementation of TCIs in practice is complex and challenged by multiple factors, particularly at the intra and inter-organizational levels. The use of theory-driven and effective implementation strategies carefully selected to address the relevant factors is highly needed to better implement TCIs. The current project provides a set of implementation strategies for this purpose. We strive for this work to be utilized by future implementers of TCIs in long-term care. As well, that it will inspire other researchers to use a similar approach for prospective efforts aiming to improve the implementation of diverse innovations in healthcare.

### Supplementary Information

**Additional file 1.** SQUIRE checklist.

**Additional file 2.** Matrices of change.

## Data Availability

Data generated and analyzed during this study are available from the corresponding author upon reasonable request.

## Abbreviations

TCIs: Transitional care innovations
CFIR: Consolidated Framework for Implementation Research
HIT: Health information technology
SQUIRE: Standards for Quality Improvement Reporting Excellence
