# Supplementary material for: Systematic development of a set of implementation strategies for transitional care innovations in long-term care
Source: Implement Sci Commun. 2023 Aug 28;4:103. doi: 10.1186/s43058-023-00487-3 (PMC10463528; doi:10.1186/s43058-023-00487-3)
Supplement: Supplementary file 2 — Additional file 2. Matrices of change. [file 43058_2023_487_MOESM2_ESM.docx]

| **Matrix 1** | | | | | | | | | | | |
| --- | --- | --- | --- | --- | --- | --- | --- | --- | --- | --- | --- |
| ***Determinants*** | | | | | | | | | | | |
| **TCIs’ use Outcomes** (*Fakha et al. 2021*)* | **Performance objectives** | ***Commitment*** | ***Organizational commitment*** | ***Leadership*** | ***Attitudes, beliefs, & motivation*** | ***Environmental conditions (structural, organizational) & professional role*** | ***Social networks*** | ***Policy*** | ***Risk perception*** | ***Feedback, reinforcement& monitoring*** | ***Innovation’s compatibility*** |
| 1) Ensure safe & timely care transitions 2) Ensure provision of continuous transitional care services 3) Reduce avoidable care transitions (e.g. hospital readmissions, institutional care) 4) Improve communication and information transfer among care providers 5) Reduce medical errors and mortality 6) Ensure patient safety 7) Improve patient satisfaction & quality of life | **Actors I: Leaders and organizations**  PO1. Ensure complexity management is performed as a TCI core component such as:   - Decide to develop individualized care plans - Decide to perform advanced care planning & comprehensive patient assessment - Support a goal-orientated and individualized delivery of transitional care - Set up a patient-centered holistic approach     PO2. Deliver care continuity as a TCI core component such as:   - Allocate for and establish staff with a transition role (e.g. advanced practice nurse, transitional care manager, health coach, care coordinator, transition care nurse) - Ensure care continuity tasks are performed by HCP (e.g. home visits, telephone follow-up, discharge planning, coordination among multidisciplinary teams, exchange of patient information, case management, finalization of long-term care arrangements) | C.1: Recognize that a TCI is important, effective, and needed C.2: Demonstrate the ability to ensure the start of and an ongoing TCI implementation | OC.1: Illustrate that TCI fits with organizational structure and goals OC.2: Express that a supportive organizational environment is important for the implementation of a TCI OC.3: Appraise that implementing a TCI is a necessary and crucial change within an organization | L1: Demonstrate an open-minded, innovative leadership style receptive to new ideas | ABM.1: Believe that engaging the key individuals is necessary for implementing a TCI ABM.2: Recognize the high benefit of implementing a TCI | EC.1: Set up health IT systems and platforms that will allow information exchange among care settings EC.2: Arrange for a certain level of resources required to implement the TCI EC.3: Create the knowledge & information platforms and channels about the TCI to facilitate the implementation EC.4: Create and organize transition roles to facilitate the TCI implementation | SN.1: Formulate work relationships and networks between the healthcare providers in different settings in order to facilitate the TCI implementation | P1: Propose policies supportive to implement the TCI | RP.1: Predict that implementing a TCI eliminates adverse risks associated with poor transitional care delivery | FRM.1: Estimate that providing feedback on a TCI implementation will enable making revisions and refinements FRM.2: Estimate that providing factual data on the TCI's benefit will support the implementation | IC.1: Recognize that designing the TCI components to match the profile of the older persons will ensure its successful implementation IC.2: Appraise the advantage of implementing a tailored TCI will improve the match between older persons' values and care |

| **Matrix 2** | | | | | |
| --- | --- | --- | --- | --- | --- |
| ***Determinants*** | | | | | |
| **TCIs’ use Outcomes** (*ref, Fakha et al. 2021)* | **Performance objectives** | ***Knowledge*** | ***Attitudes & beliefs*** | ***Motivation*** | ***Skills*** |
| 1) Ensure safe & timely care transitions 2) Ensure provision of continuous transitional care services 3) Reduce avoidable care transitions (e.g. hospital readmissions, institutional care) 4) Improve communication and information transfer among care providers 5) Reduce medical errors and mortality 6) Ensure patient safety 7) Improve patient satisfaction & quality of life | **Actors II: Healthcare professionals**  PO1. Engage the patient & caregiver as TCI core components such as:   - Establish trusting relationships with patient and caregiver - Understand patient's goals & preferences - Enable collaboration between patient, family caregivers, and primary care providers   PO2. Educate the patient & caregiver as TCI core components such as:   - Conduct discharge planning using “teach-back” methods with patient - Coach the patient in:   ○ medication self-management  ○ using a patient-centered health record  ○ knowledge of “red flags”  ○ making primary care provider/specialist appointments   - Provide patient/caregiver education tools - Coordinate education & community services to - develop self-management skills of the patient   PO3. Provide complexity management as a TCI core component such as:   - Develop individualized care plans - Carry out advanced care planning & comprehensive patient assessment - Apply a patient-centered holistic approach   PO4. Provide care continuity as a TCI core component such as:   - Execute the duties of a transition role professional - Conduct care continuity tasks (e.g. home visits, telephone follow-up, discharge planning, coordination among multidisciplinary teams, exchange of patient information, case management, finalization of long-term care arrangements) | K.1: Describe the components of the TCI K.2: Recall the tasks, steps, & procedures required to implement the TCI | AB1: Express positive attitude towards the TCI as an innovation  AB2: Believe that the TCI is beneficial to enhance transitional care for older persons | M1: Express interest and intention to implement the TCI | S1: Demonstrate the ability to deliver the components of the TCI  S2: Demonstrate the ability to navigate and adhere to the TCI's implementation tasks |

**Reference: Fakha A, Groenvynck L, de Boer B, van Achterberg T, Hamers J, Verbeek H. A myriad of factors influencing the implementation of transitional care innovations: a scoping review. Implement Sci. 2021;16(1):21.*
